# Stock movement prediction in a hotel with multimodality and spatio-temporal features during the Covid-19 pandemic

**DOI:** 10.1016/j.heliyon.2024.e40024

**Published:** 2024-11-03

**Authors:** Yang Liu, Lili Ma

**Affiliations:** a School of Information Management, Wuhan University, 430072, China; b Economics and Management School, Wuhan University, 430072, China

**Keywords:** Stock movement prediction, Multimodal data, Spatio-temporal feature, Graph convolutional network, Covid-19 pandemic

## Abstract

The COVID-19 pandemic has underscored the importance of accurate stock prediction in the tourism industry, particularly for hotels. Despite the growing interest in leveraging consumer reviews for stock performance forecasting, existing methods often need to integrate the rich, multimodal data from these reviews fully. This study addresses this gap by developing a novel deep learning model, the Multimodal Spatio-Temporal Graph Convolutional Neural Network (MSGCN), specifically designed to predict hotel stock performance. Unlike traditional models, MSGCN captures the spatial relationships between hotels using a graph convolutional network and integrates multimodal information—including text, images, and ratings from consumer reviews—into the prediction process. Our research builds on existing literature by validating the efficacy of multimodal data in improving stock prediction and introducing a spatio-temporal component that enhances prediction accuracy. Through rigorous testing on two diverse datasets, our model demonstrates superior performance compared to existing approaches, showing robustness during and after the COVID-19 pandemic. The findings provide valuable insights for hotel managers and consumers, offering a powerful tool for making informed business decisions in a rapidly evolving market.

## 1 Introduction

The outbreak of the pandemic caused extreme volatility in global financial markets. It has fundamentally affected the global economy as a public health emergency [1]. Goodell [2], reported that the COVID-19 pandemic has had a greater impact on the stock market than any previous infectious disease outbreak. As a result, the inevitable travel, trade, and hotel reduction severely affect global economic activity. Especially in the hotel industry, which is highly dependent on the market, the long-term impact of COVID-19 remains, with reduces consumer demand for hotel services due to health and hygiene precautions [3]. Hence, examining how the hotel and tourism industry must prepare for severe economic crises, such as the COVID-19 pandemic is necessary.

Today, the digital age has made it easier for consumers to exchange information, which has led to an explosive increase in on-line reviews. More hotels adopt consumer reviews to understand their sentiment, which has important implications for the financial performance of hotels. The literature [4] shows that positive consumer sentiment can fuel the growth of hotel stocks. Moreover, with the development of artificial intelligence, machine learning technology has been widely applied to consumer sentiment analysis [5]. This method mainly focuses on the emotional characteristics of consumers, ignoring descriptive information. In addition, previous machine learning methods only focused on unimodal emotion [6], but ignored multimodal emotion. The proposal of multimodal deep learning can enrich the feature extraction of consumer reviews [7], promoting hotel stock performance [8].

This study demonstrates hotel reviews using advanced multimodal deep learning, which can provide strategic advice for hotel practitioners. Hoteliers must weigh the economic impact directly attributable to the pandemic in the hospitality industry during COVID-19. Economic policies affect support for any initiative: on the one hand, understanding the effects of the pandemic on the sector helps hoteliers to address effective decision-making frameworks, which ensures rapid responses to contingencies that challenge the solvency of their businesses. Hoteliers’ investment decisions require accurately determining the direction of their economy and the stock market. Furthermore [9], demonstrated a positive relationship between consumer sentiment and firm performance, and [10] proved that consumer reviews could predict corporate stock market performance.

Therefore, using consumer reviews to predict hotel stocks can help investors optimize their decisions by providing real-time insights into customer sentiment, preferences, and emerging trends [11]. This approach allows for a more nuanced understanding of market dynamics, especially during periods of significant disruption such as the COVID-19 pandemic. By analyzing these reviews, investors can gain a clearer picture of how consumer behaviour and expectations are evolving, which in turn can inform more strategic investment choices. On the other hand, the hospitality industry, along with other sectors, has faced the dual challenge of navigating a severe public health crisis and coping with a sharp decline in economic activity. These challenges have forced companies to adapt quickly, employing various market responses to mitigate losses and maintain operations. This includes strategies like altering pricing models, enhancing hygiene standards, and shifting to digital platforms to meet changing consumer demands. By evaluating these factors, industry stakeholders can better understand how different elements—such as government regulations, consumer confidence, and supply chain disruptions—interact to influence the hospitality sector's recovery and long-term sustainability. This comprehensive assessment is crucial for identifying the most effective strategies to support the industry's various facets, from hotel operations to customer engagement, in the post-pandemic landscape.

This paper assesses the multimodal information in consumer reviews by pre-trained models from two data sources. Furthermore, the location information of hotel stores is constructed using a multimodal spatio-temporal graph convolutional neural network (MSGCN) based on the use of a graph neural network (GNN) [12]. The time-series information of text is acquired through long short-term memory (LSTM) [13]. Finally, this framework can predict the hotel's stock price. This paper explores the interactive relationship between consumer sentiment and hotel stock prices, which expands the theoretical boundary of consumer sentiment factors. This research also provides new solutions for managers to deal with financial performance decision management under COVID-19. This paper makes several contributions to the literature on hotel stock forecasting. First, a new deep learning model is proposed, which uses spatio-temporal characteristics to generate multimodal features. This model has solid computational capabilities and good applicability to various travel platforms. Second, a more scientific and effective feature extraction method, namely the convolution operation, is used to capture emotional information that is highly implicit in consumer reviews. Third, the representation of spatial relationships based on multimodal data contributes to increased predictive accuracy.

The tourism and hospitality sectors are susceptible to market fluctuations, crises, and consumer sentiment. Understanding the factors influencing stock performance, particularly in a post-pandemic world, helps industry professionals better navigate uncertainty and make informed decisions. By integrating consumer reviews, which include text, images, and ratings, our research highlights how multimodal data can provide more comprehensive insights than traditional financial metrics alone. This is particularly relevant in the digital age, where consumer feedback can strongly influence market trends. The pandemic has drastically changed consumer behavior and market dynamics, making traditional forecasting models unreliable. Researching this topic offers insight into how new methods can adapt to evolving conditions, enhancing prediction accuracy and providing actionable guidance for stakeholders. For hotel managers, investors, and policymakers, accurate stock predictions offer a strategic advantage in optimizing operations, managing risks, and making investment decisions. By understanding the research, these groups can apply cutting-edge models like MSGCN to enhance their business strategies.

The paper is structured as follows: Section 2 begins with a review of the literature in three sections; Section 3 presents our proposed model. Our analytical framework includes data pre-processing, multimodal deep learning, and visual analytics to achieve this. This research in Section 4 then describes experiments with our proposed model and compares it to existing algorithms. Section 5 presents the results of our experiments and illustrates the advantages of our approach. Finally, the main findings, decision-making implications, and future research directions are given in Section 6. Morover, we give the conclusions in Section 7.

## 2 Literature review

### 2.1 COVID-19: the impact on hospitality industry performance

The COVID-19 pandemic has had a significant adverse impact on the hospitality industry. Individual hotels and major operators are forecasting occupancy rates below 20 %. Hao et al. [14] reviewed the overall impact of the pandemic on Chinese tourism. The result is a significant drop in industry hotels, and a loss of more than $9 billion in revenue. Crespí-Cladera et al. [15] utilized a stress approach to estimate potentially poor performance in Spanish hotel companies. They also demonstrated that nearly 25 % of sample companies faced a 60 % reduction in financial distress. They also found that most of these companies would also face solvency issues. More specifically, they note that Spain's total number of hotel nights fell from 184.7 million in the first seven months of 2020. Finally, Clark et al. [16] estimated the negative average cumulative abnormal return to 17.54 % from 54 public hotel companies in 23 countries. Significantly, COVID-19 has directly affected the performance of the hospitality industry.

The stock market has exhibited unusual stock declines and volatility during a pandemic shock far worse than any previous economic crisis. The pandemic's cause, scope, and damage remain uncertain [17]. COVID-19 directly limited the company's financial risk to the company's economic activity [18]. Investors have been worried about the long-term loss of income, which damages firms' financial liquidity and their investments [19]. For example, Baker et al. [20] revealed the government's renewed strict restrictions on business activity caused US stock markets to drop more than in previous pandemics. Considering that the hospitality industry is regarded as one of the hardest-hit industries due to COVID-19, the current research assumes that COVID-19 harms the stock returns of travel and hospitality companies worldwide.

While the existing literature extensively examines the impact of COVID-19 on the tourism industry, including its devastating effects on travel patterns, hotel occupancy rates, and overall industry revenue, it falls short in addressing a crucial aspect—how consumer sentiment, mainly as reflected in online reviews, can influence hotel stock prices. The emotional characteristics expressed in these reviews, such as satisfaction, frustration, or disappointment, could serve as vital indicators for stock market performance in the hospitality sector. Despite recognizing consumer sentiment as a powerful tool for predicting market trends in other industries, there must be a noticeable gap in applying this concept to the hotel sector, especially during the pandemic. This presents a significant opportunity for future research to bridge this gap by analyzing the correlation between online consumer sentiment and hotel stock performance during and after the COVID-19 crisis.

### 2.2 Studies of hospitality stock performance

The research into hotel finance is divided into macroeconomic and behavioral financial factors. On the one hand, macro-factors are market index models that decompose into systematic risk and firm-specific risk, which asserts that macro-factors influence the returns of market portfolios [21]. On the other hand, multifactor models allow stock returns to be proxied by some macro-variables, which promise to provide a better return. Currently, Kot et al. [22] examined short-selling activities in the hotel industry, testing results by periods and individuals, and the industry shows that the supply side is very similar. The heavily shorted hotel stocks will underperform in the year ahead. Esen et al. [23] suggested that external investors may do well by imitating insider trading. In addition, insider buying was found to have a higher abnormal return than insider selling predictions after the event.

A second factor is that consumer sentiment affects hotel stock returns, which extends to behavioral finance. Moulton et al. [24] found that non-hotel companies distract investors, reducing hotel stock reactions to earnings news, and they are turning attention to behavioral finance. Chen et al. [25] found adverse momentum effects on Taiwanese hotel stocks in the short to long-term, and the mid-range is in the middle of various hotel performances. The consumer sentiment analysis discussed in the existing literature often overlooks the emotional nuances embedded in online reviews, leading to a limited understanding of the true sentiments expressed by consumers. Most of these studies focus on superficial sentiment categorization, neglecting the underlying emotional factors that can provide deeper insights into consumer attitudes and behaviors. To address these limitations, multimodal deep learning approaches are being adopted to extract implicit sentiment from consumer reviews. Unlike conventional methods, multimodal deep learning integrates various data types—text, images, and audio—to capture a more comprehensive view of consumer sentiment. Doing so can uncover subtle emotional cues that traditional methods might miss, offering a richer and more accurate sentiment analysis. This advanced approach enhances the understanding of consumer emotions. It provides valuable insights that can be used to predict consumer behavior and, by extension, influence market dynamics, such as hotel stock prices.

### 2.3 Machine learning in the hotel management literature

Machine learning has already been used in analyses of hotel management, Kirilenko et al. [26] examined how tourism research can be divided into two parts: sentiment analysis and predictive consumer recommendation decision. Sentiment analysis or opinion mining generally uses machine learning, ML, and natural language processing [5]. Sentiment analysis utilizes models to determine consumer sentiment toward a product or service. Sentiment analysis is mainly divided into two aspects: aspect sentiment and sentiment classification. Lim et al. [27] utilized regression to explain the importance of expectations to consumers. Lee et al. [28] used the help of machine learning to identify consumer satisfaction in on-line reviews. Over the past few years, many researchers have included consumer recommender systems to measure consumer satisfaction. They found that business growth is proportional to the net promoter score. Consumer recommendation value is regarded as the basis of consumer satisfaction. Siering et al. [29] predicted opinion date decisions in airline consumer recommendations using qualitative content from on-line revisits, while [30] employed quality to predict static and quantitative content revision decisions in consumer recommendations. Contessi et al. [31] presented a two-step approach utilizing historical and advanced booking data; empirical results demonstrate the superiority of PCA-based methods across all hotels and forecasting horizons.

Nowadays, deep learning has been widely used in the travel industry. Li et al. [32] proposed spatio-temporal fusion on both GCN and LSTM, and the results show that combining spatial effects can significantly reduce forecast errors. Deep learning techniques are mainly used for computer vision and image processing [33,34] and text analysis [35]. In other words, multimodal-based deep learning remains in its infancy. Cheng et al. [36] proposed using advanced techniques such as deep learning to help tourism research obtain more in-depth analysis of data about tourist attractions from different perspectives. Therefore, only some studies elucidate the application of multimodal deep learning to the hotel industry, especially in hotel stock forecasting. Thus, multimodal deep learning was adopted in the present work to give hotel practitioners strategic insights into finance.

Recently, Chen et al. [37] introduced a directed graph convolutional network (GCN) to extract text features. Xiang et al. [38] utilized a GCN to aggregate node-to-node parts, which captures semantic contextual information. However, despite the advancements in sentiment analysis using multimodal deep learning, these methods still fail to capture the need to capture the spatial-temporal characteristics unique to each hotel. The current approaches primarily focus on extracting sentiment from consumer reviews without considering the influence of a hotel's geographic location, time-specific factors, or the dynamic interactions between these elements. This omission limits the ability to fully understand and predict the impact of consumer sentiment on a hotel's stock performance. To address this gap, the present work introduces GCN to enhance the analysis by capturing multimodal interaction information and integrating spatio-temporal features [39]. By incorporating GCN, the analysis captures the nuanced sentiment expressed in consumer reviews. It considers how these sentiments are influenced by the hotel's location and temporal factors, such as seasonal trends or local events. This comprehensive approach enables a more accurate prediction of hotel stock performance, reflecting the real-world complexities that traditional methods might overlook.

## 3 Methodology

### 3.1 Task model

**Fig. 1:**
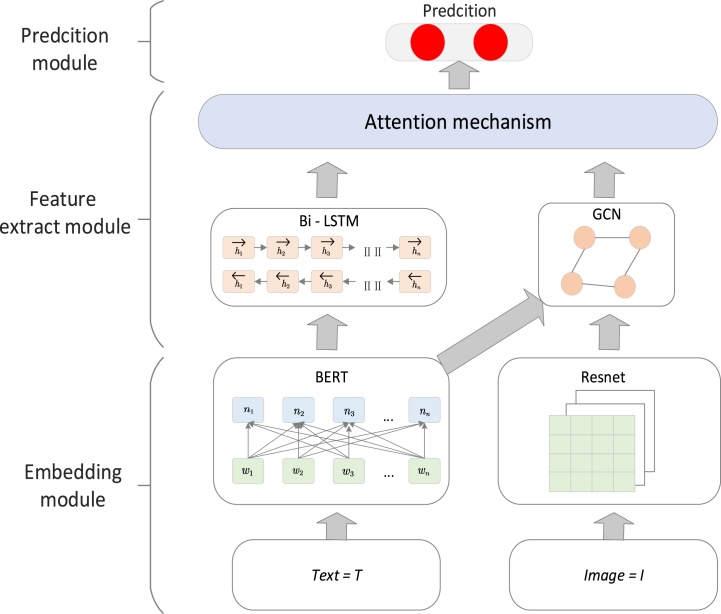
Caption: Architecture of the MSGCN model.

Given a consumer review where the text is *t*, the image is *i*, and the stock of the hotel is the target variable, our task is to predict the movement of the stock (up or down), where $y_{i}$ represents the expected outcome. Fig. 1 illustrates our proposed model, MSGCN. This model is specifically designed to leverage the rich, multimodal data provided by consumer reviews, integrating both textual and visual information to make more accurate stock movement predictions. The MSGCN model is composed of three core components:

Embedding Module: This module plays a critical role in the model by mapping the multimodal data, including both the text *t* and image *i*, to dense token embeddings. These embeddings serve as a foundational representation of the input data, capturing the semantic and visual features necessary for downstream processing. The embedding module ensures that both the textual and visual information are represented in a compatible format, allowing for effective integration and analysis [10].

Feature Extraction Module: After the data is embedded, the feature extraction module processes these embeddings to capture deeper, more complex patterns within the data. This module employs a combination of bidirectional Long Short-Term Memory (BiLSTM) networks and Graph Convolutional Networks (GCN). The BiLSTM component is used to capture sequential dependencies and contextual relationships within the text data, while the GCN is applied to model the interactions and dependencies between different elements of the multimodal data, enabling the extraction of both local and global features [40]. Together, these techniques ensure that the model effectively captures the intricate relationships between the textual and visual content, which are crucial for accurate prediction.

Prediction Layer: The final component of the MSGCN model is the prediction layer, which is responsible for generating the final output $y_{i}$ , indicating the predicted stock movement. This layer consists of a linear transformation that takes the extracted features and maps them to a binary outcome, representing either an upward or downward movement in the hotel stock. The design of the prediction layer is optimized to ensure that the features extracted by the previous modules are effectively utilized to produce accurate and reliable predictions.

The subsequent sections of this paper will provide a detailed description of each of these components, including the specific architectures and algorithms used, as well as the rationale behind their design choices [41]. Additionally, we will discuss the training process and the evaluation metrics used to assess the performance of the MSGCN model, demonstrating its effectiveness in predicting stock movements based on multimodal consumer review data.

### 3.2 Embedding layer

#### 3.2.1 Text by BERT

The BERT model is adopted to obtain the word representations [42], which is a pretrained model that can enrich the feature dimension of words. The sentence with each token is extended to length *r*, where *n* is the maximum length of the sentence in the mini-batch. The average pooled embedding of the corresponding token in the BERT layer is represented as the token representation, it can be expressed as sequence *H =* ($h_{1},h_{2}$, …, $h_{n}$), which was launched by Google's open-source warehouse TensorFlow Hub. The output of the pretrained model can be divided into two types: Pooled_output and Sequence_output. Sequence_output forms its corresponding feature vector for each word in the input sentence.

First, this model defines the output vector matrix as $W_{s}\in R^{m\times n\times d_{e}}$, where *m* is the number of elements in the text, *n* denotes the number of words contained in each element, *d_e* represents the dimension of word embedding, *w_e* in the matrix corresponds to the vector output of the *e*th word in the *i*th, then the text vector output is:

$$W_{s}=\left[\begin{array}{c} \left[w_{1}^{1},w_{2}^{1},\ldots ,w_{n-1}^{1},w_{n}^{1}\right]\\ \left[w_{1}^{2},w_{2}^{2},\ldots ,w_{n-1}^{2},w_{n}^{2}\right]\\ \vdots \\ \left[w_{1}^{m},w_{2}^{m},\ldots ,w_{n-1}^{m},w_{n}^{m}\right] \end{array}\right]$$

The Pooled_output mode pools the word vectors in each list element, which summarizes them into a vector. The output text vector is in the following form, where $w_{i}$ represents the sentence vector of the *i*th element in the text list:

$$W_{s}=\left[w_{1},w_{2},\ldots ,w_{n}\right]$$

#### 3.2.2 Image embedding by resnet

**Fig. 2:**
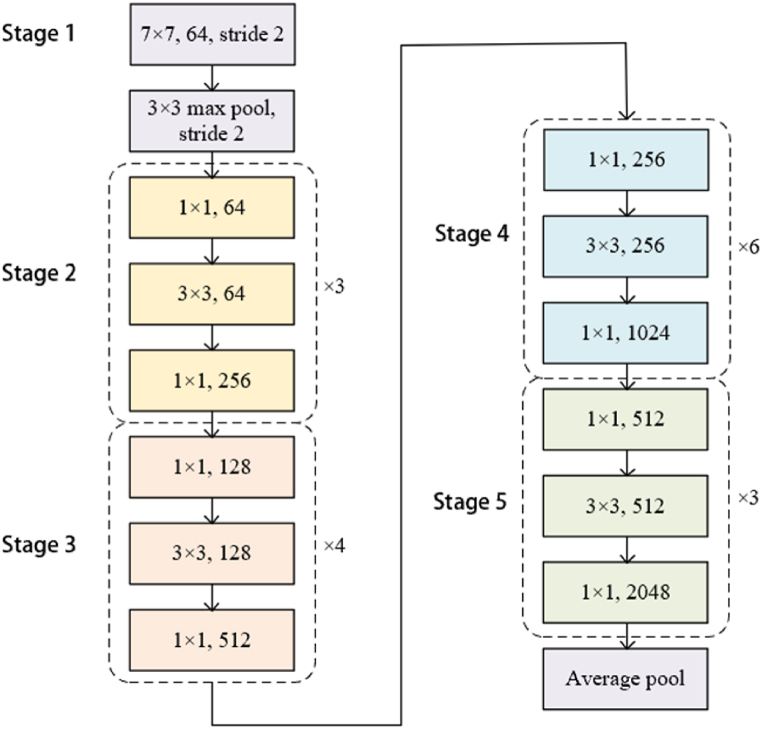
Caption: The pre-training process of the image.

This paper adopts ResNet50 as the image embedding model on consumer reviews, and its main structure consists of 49 convolutional layers and one fully connected layer. The model generally divides ResNet50 into five stages. As shown in Fig. 2, the structure of the first stage is regarded as the pre-processing of the input, and the remaining four stages are composed of bottleneck modules. The second stage includes three bottlenecks. Herein, the image decoder extracts the picture code, and the color value of each pixel in the RGB three channels is obtained through a three-dimensional vector with a size of 338 × 338 × 3. The three-dimensional vector is then passed into the pre-trained model of ResNet50, and the image features with dimension 2048 are output.

**Fig. 3:**
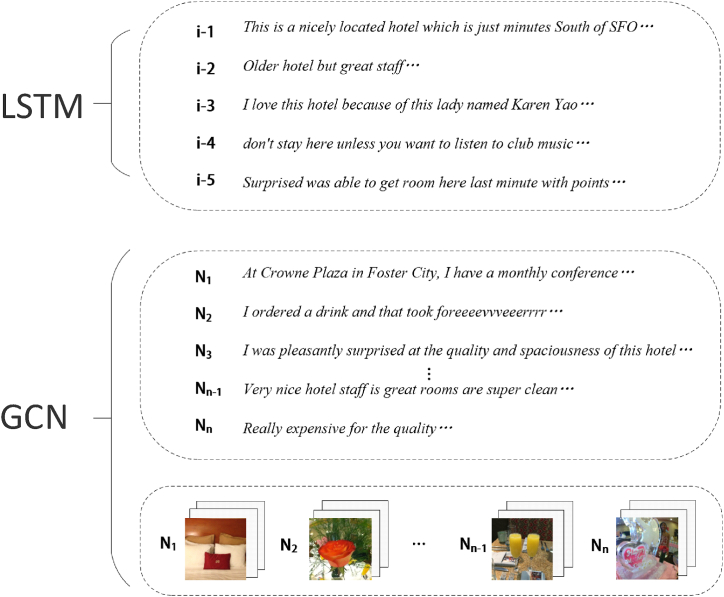
Caption: The input of text and images.

### 3.3 Feature extract modules

This module mainly revolves around feature extraction of multimodal data. Fig. 3 shows the input module of text and pictures. The text is input to LSTM, and the picture is input to GCN. This research also defines text as *i* and image as *n*.

#### 3.3.1 LSTM

LSTM is a recurrent neural network that processes time-series data. LSTM introduces a gate mechanism to control each neuron's retention of historical neuron information the better to integrate historical information. LSTM captures remote dependencies sequentially via memory cell and gate mechanisms. The LSTM calculation is presented as follows:

$$f_{t}=\sigma \left(W_{f}∙\left[h_{t-1},x_{t}\right]+b_{f}\right)$$

$$i_{t}=\sigma \left(W_{i}∙\left[h_{t-1},x_{t}\right]+b_{i}\right)$$

$$\tilde{C_{t}}=\tanh\;\left(W_{c}∙\left[h_{t-1},x_{t}\right]+b_{c}\right)$$

$$C_{t}=f_{t}*C_{t-1}+i_{t}*\tilde{C_{t}}$$

$$O_{t}=\sigma \left(W_{o}\left[h_{t-1},x_{t}\right]+b_{o}\right)$$

$$h_{t}=o_{t}*\tanh\;\left(C_{t}\right)$$

where σ represents the logistic sigmoid function, $i_{t}$, $f_{t}$, and $C_{t}$ mean input gate and forget gate, respectively. The output gate at time *t*, and the memory unit activation vector have the same dimensions as the hidden layer vector $h_{t}$. The *W* and *U* weight matrices are model parameters in the LSTM.

These data are aggregated as input for date *i.* Assuming a given date *i* for the LSTM network, five date-time points in its history (*i* − 5 to *i* − 1) are recorded, which are used as input for date *i.* All texts within the last month of the date *i* are also determined, which can be summarized as multidimensional data.

The word sequence adopts a bidirectional LSTM to encode the word and obtain the hidden layer representation $h_{in}$ at the end of the time step. The representation of the word *i* is demonstrated through concatenation of $\vec{h_{ni}}$ and $\overset{⃖}{h_{ni}}$ ,

$$\vec{h_{in}}=\vec{LSTM}\left(x_{i}\right),i=\left[1,L\right]$$

$$\overset{⃖}{h_{in}\;}=\overset{⃖}{LSTM}\left(x_{i}\right),i=\left[L,1\right]$$

$$h_{in}=\left[\vec{h_{in}\;},\overset{⃖}{h_{in}\;}\right]$$

#### 3.3.2 GCN

The GCN summarizes the features of the node itself and its neighbors with edges. Generating a new feature representation is a node feature processor acting on the topological graph. The GCN can use the fixed local edge structure of the image with each training iteration, which updates the nodes of the entire structure of the model. Moreover, all nodes of the GCN share the filter parameters, reducing the computational burden.

The initial feature vector *X* of all nodes, the vector dimension is *N* × *D*, where *N* represents the number of nodes, *D* denotes the feature dimension of each node. In the adjacency matrix *A* of the node, the matrix dimension is *N* × *N*. The updating function is as follows:

$$H^{\left(l+1\right)}=\sigma \left({\tilde{D}}^{-\frac{1}{2}}\tilde{A}{\tilde{D}}^{-\frac{1}{2}}H^{\left(l\right)}W^{\left(l\right)}\right)$$

where $\tilde{A}$ is the sum of the adjacency matrix *A* and the identity matrix; $\tilde{D}$ is the degree matrix of $\tilde{A}$. *H* corresponds to the node features of each layer, then $H_{0}$ = *X*.

Here, the image is only input into the GCN, and this method is the same as in the previous processing method, all the text and images in the previous month are obtained, then classified according to the nodes of the GCN. The GCN extracts the feature on the graph *G =* (*V, E*), its input includes two parts: the node feature *F* and the adjacency matrix *A*. The feature $f_{i}$ is the text feature vector output in each node *i* for BERT. The feature description of all nodes expresses as an *N×D* feature matrix *F*, where *N* represents the number of nodes, *D* is the dimension of text features. The adjacency matrix *A* maps in the form of a vector structure of size *N×N*. Each $A_{i,j}$ represents the edge weight of the node $N_{i}$ and node $N_{j}$, the adjacency matrix *A* is a symmetric matrix.

Here, the nodes of the graph structure represent the cities. The feature matrix is aggregated according to the text features of different locations, and the edge connection weight of the adjacency matrix, which measures the cosine similarity through the latitude and longitude $L_{i}$ of the city $C_{i}$, and is expressed as:

$$A_{i,j}=similarity\left(C_{i},C_{j}\right)=\frac{L_{i}∙L_{j}}{\left\|L_{i}\right\|\times \left\|L_{j}\right\|}$$

A global pooling layer is superimposed after the GCN layer to pool the features of all nodes, and the features are pooled to achieve the purpose of dimensionality reduction.

#### 3.3.3 Attention mechanism

The feature vectors of text and images are integrated into the attention mechanism. A corresponding attention score is obtained by a non-linear item. Each attention score is then normalized by use of a softmax function. The final representation for the prediction label is *y*, which is the weighted average of all $z_{i}$.

$$u_{i}=\tanh\left(W_{i}Z_{i}+b_{i}\right)$$

$$\alpha_{i}=\frac{\exp\;\left(u_{i}\right)}{\sum_{l=1}^{m}\;\exp\;\left(u_{i}\right)}$$

$$y_{i}=\sum_{i=1}^{m}\alpha_{i}z_{i}$$

### 3.4 Prediction modules

In this study, binary cross-entropy is used for model optimization:

$$Loss=-\frac{1}{output\;size}\sum_{i=1}^{output\;size}y_{i}∙\log\hat{y_{i}}+\left(1-y_{i}\right)∙\log\;\left(1-\hat{y_{i}}\right)$$

## 4 Experimental setting

### 4.1 Data collection

#### 4.1.1 Consumer review data

The top four cities (New York, Miami, San Francisco, and Los Angeles) are selected based on this ranking of the total number of tourists in the USA. We likely ranked U.S. cities according to the total number of tourists they attract annually. This ranking is essential because it helps identify the most relevant and high-impact markets within the hospitality industry, where fluctuations in consumer sentiment influence hotel stock performance. Our goal is to analyze reviews in English. Therefore, Yelp and TripAdvisor are merchants with popular travel websites. There are restaurants, shopping malls, hotels, tourist venues, and others reviewed thereon; this article uses the Python-based selenium automation tool to review the Yelp website and TripAdvisor for four hotel brands (Crown Plaza, Hilton, Marriot, and Westin). These cities were chosen because they have large volumes of visitors, a significant number of hotels, and substantial market activity that can provide meaningful data for analysis. These urban centers also represent diverse geographic locations across the U.S., which allows the study to account for regional differences in consumer behaviour and market dynamics. These hotel chains are known for attracting a high volume of tourists and business travellers, making them ideal candidates for studying the impact of consumer sentiment on stock performance [43]. Additionally, these brands operate across various market segments—from mid-range to luxury—allowing the study to capture a broad spectrum of consumer sentiment and its potential impact on stock prices. Their corresponding review attributes are crawled, and the distribution of the number of stores and reviews corresponding to each hotel brand is summarized in Table 1.

**Table 1:** Data information of review corpus in two cases of the hotel.

Data source	Hotel	Number of stores	Consumer reviews
TripAdvisor	Crown Plaza	35	8596
	Hilton	28	7835
	Marriot	43	8247
	Westin	34	7952
Yelp	Crown Plaza	26	6851
	Hilton	59	8463
	Marriot	27	5786
	Westin	18	6245

The data are divided into two time-periods: before March 2020 and after March 2020 based on the COVID-19 pandemic. Table 2 summarizes the partition information of the two datasets.

**Table 2:** Detailed division of each dataset.

Data source	Hotel	Period	Time span		Number	
			Before COVID-19	After COVID-19	Before COVID-19	After COVID-19
TripAdvisor	Crown Plaza	12.5.2011–7.10.2121	12.5.2011–29.2.2020	1.3.2020–7.10.2121	7025	1544
	Hilton	18.5.2011–15.10.2121	18.5.2011–29.2.2020	1.3.2020–15.10.2121	6630	1205
	Marriot	3.4.2011–18.10.2021	3.4.2011–29.2.2020	1.3.2020–18.10.2021	6784	1463
	Westin	4.12.2011–16.10.2021	4.12.2011–29.2.2020	1.3.2020–16.10.2021	6846	1106
Yelp	Crown Plaza	8.6.2012–8.10.2121	8.6.2012–29.2.2020	1.3.2020–8.10.2121	5647	1204
	Hilton	4.5.2012–8.10.2121	4.5.2012–29.2.2020	1.3.2020–8.10.2121	6889	1575
	Marriot	7.5.2011–15.10.2021	7.5.2011–29.2.2020	1.3.2020–15.10.2021	4421	1365
	Westin	8.5.2011–10.10.2021	8.5.2011–29.2.2020	1.3.2020–10.10.2021	4768	1486

#### 4.1.2 Stock data

In the present work, the stock movement of each hotel group based on the US stock market is undertaken. Since Westin and Marriott both belong to the Marriott Group, the stock dataset consists of the stock data for three companies: the Marriott International Hotel Group, the Hilton Hotel Group, and the InterContinental Hotel Group.

The source of the stock data set is Yahoo Finance (https://finance.yahoo.com), which provides the historical data interface of the stock in Table 3. These data cover the fundamental stock indicators of the stock daily since its first listing (in the opening, high-low, and close format), with adjusted closing price and volume listed. This article divides the training set and test set according to the ratio of 8:2. The total number of items in Crown Plaza before the epidemic was 7025, its training set was 5620, and its test set was 1405.

**Table 3:** Stock quotes.

Hotel	Stock symbol	Number of matching reviews
Crown Plaza	InterContinental Hotels Group PLC (IHG)	4745
Hilton	Hilton Worldwide Holdings Inc. (HLT)	4115
Marriot	Marriott International, Inc. (MAR)	6016
Westin	Marriott International, Inc. (MAR)	6016

### 4.2 Data pre-processing

Since the corpus in the consumer reviews is open, the text contains much noisy information. The data needs cleaning before constructing the time-series text data. First, the non-text content that cannot be recognized in the text corpus is deleted, including garbled characters, emoticons, and punctuation marks. Secondly, according to the stop words list in the NLTK, some common words that do not have specific semantics are stopped to improve the efficiency and generalization ability of the subsequent model.

The stock-price prediction problem is simplified to a two-category problem. The prediction label of a specific date is defined as $L_{i}$. The opening price and closing price of the day are $P_{i,open}$ and $P_{i,close}$, respectively.

$$L_{i}=\left\{ \begin{array}{c} 1,{\;P}_{i,open}<\;P_{i,close}\\ 0,{\;P}_{i,open}\geq P_{i,close} \end{array}\right.$$

Synchronously, the opening dates of the US stock market are not continuous: stock label values of the missing dates are supplemented to ensure continuity. The specific operation involves synchronizing the data of the blank date as the label of the first opening day after the date. However, there are differences in the period of the release time of review and stock data. On the one hand, some studies are released earlier than the listing time. On the other hand, the earliest reviews of hotels are released much later than the listing time of the hotel (herein, the intersection of the two-time spans is retained and data from other periods deleted).

This study focuses on a select group of cities and specific hotel chains. This selection may introduce sampling bias, as it does not represent the entire hospitality industry or other geographic regions with different consumer behaviours and market dynamics. To mitigate this, we carefully selected these cities and hotels based on their high tourist traffic and prominence in the U.S. market. This ensures that the data collected is from high-impact areas, although the findings may only partially generalize to smaller markets or less prominent hotel brands. The data collection may be influenced by specific periods, such as peak tourist seasons or during particular events (e.g., holidays, festivals), which could skew the sentiment analysis towards either overly positive or negative sentiments. The study includes data from various times of the year to capture a more balanced view of consumer sentiment. By analyzing data across different seasons and periods, we aim to reduce the impact of temporal bias and provide a more accurate representation of overall sentiment trends.

### 4.3 Performance evaluation

Proper parameter setting is the key to better predicting stock performance. The main parameters of the model include: the number of hidden units, time step size, learning rate, training period, and batch size. This research focuses on the effects of time step size and number of hidden units on prediction performance. The best combination of parameters ∈ {1, 2, ⋯,12} and units ∈ {1, 4, 8, ⋯,256} is selected from the time step in the exhaustive grid search method on each technical criterion. In addition, the learning rate, training period, and batch size are manually adjusted and set to 0.001, 200, and 32, respectively. This work follows the commonly used evaluation metrics, including accuracy = (TP + TN)/(TP + FP + FN + TN), where TP, FN, FP, TN respectively denote the number of true positive, false negative, false positive, and true negative samples.

## 5 Results

### 5.1 Data in TripAdvisor

Overall, the model in this paper outperforms traditional machine learning and deep learning, and the model's superiority indicates spatial effects in the prediction of stocks in Table 4. These effects are beneficial to enriching the feature extraction of the model. This work is based on the best performance in terms of accuracy; more specifically, the model is more accurate than other models.

**Table 4:** Holistic comparison of different hotels and different algorithms in TripAdvisor, the performance is measured based on accuracy, these values are a percentage (For example, the higher the better).

	Before COVID-19				After COVID-19			
	Crown Plaza	Marriott	Westin	Hilton	Crown Plaza	Marriott	Westin	Hilton
LR	50.25	51.58	49.94	51.58	49.63	50.77	49.03	50.96
DT	51.95	50.56	51.48	50.46	50.56	50.14	51.89	50.02
SVM	50.87	49.23	48.52	50.25	49.36	49.08	50.63	49.10
AB	52.14	51.96	52.07	51.86	51.85	51.55	51.69	50.17
XGB	52.74	51.87	52.56	51.52	51.69	51.08	52.35	50.56
CNN	54.63	55.32	55.14	54.09	53.05	54.72	54.93	51.74
LSTM	55.47	56.63	56.24	55.74	55.21	55.97	55.47	55.02
MSGCN	59.25	60.25	60.74	59.54	59.02	59.41	60.18	59.08

Deep learning is shown to be better than traditional machine learning by a few percentage points on average (*such as,* 60.74 % for Westin and 60.25 % for Marriott). First, the model can consistently outperform other models in all prediction scenarios, and simple models can produce the best prediction results. Firstly, the performance in deep learning is much better, especially for Westin and Hilton because these two hotels are larger and have more complete data. In addition, the deep learning model is part of the model proposed in this paper and it performs better. Second, the LSTM can better capture non-linear and complex time-series data. Compared with traditional machine learning, LSTM results in an improvement of 2 %, and if accurate stock prediction is achieved by a single model, then LSTM model is a good choice. Third, GCN operations can better capture the spatial information between tourist destinations instead of simply multiplying the spatial relationship matrix.

Both GCN-based models outperform traditional deep learning and machine learning, promising improvements in comparison. Fourth, a more flexible description of the spatial relationships of hotels improves the hotels, respectively. This result confirms the adopted requirement to predict improved similarity maps, confirming the spatio-temporal extent, so the gap is also narrowing.

### 5.2 Data in yelp

Table 5 shows that our model is superior and outperforms other models. Specifically, our model outperforms traditional machine learning and the previously processed data and, by and large, performs in a manner aligned with the previous data with a similar mean. This result indicates that the present model outperforms two datasets, suggesting both are useful.

**Table 5:** Holistic comparison of different hotels and different algorithms in in Yelp, the performance is measured based on accuracy, these values are a percentage (For example, the higher the better).

	Before at COVID-19				After at COVID-19			
	Crown Plaza	Marriott	Westin	Hilton	Crown Plaza	Marriott	Westin	Hilton
LR	51.23	51.69	51.20	50.89	50.41	50.90	50.78	50.12
DT	50.85	50.46	50.78	50.66	50.46	50.23	49.41	49.90
SVM	52.36	51.88	52.01	53.16	51.45	50.49	51.79	52.82
AB	54.36	53.46	53.71	54.85	53.74	52.89	52.94	53.97
XGB	55.31	56.24	55.85	55.76	56.03	56.04	55.13	54.79
CNN	56.12	56.78	55.47	56.89	55.25	55.14	54.63	55.14
LSTM	56.32	56.85	56.71	55.46	55.46	56.13	56.41	54.26
MSGCN	59.57	59.23	60.13	59.78	58.12	58.36	59.16	58.16

In particular, the pre-COVID-19 performance is significantly better than the post-COVID-19 performance (by several percentage points); in addition, all spatio-temporal models outperform the four single models, indicating that combining spatial effects can improve prediction accuracy. The conclusion suggests that our model can provide optimal prediction results in both short and long-term forecasting. The proposed model remains valid for stock prediction during the COVID-19 pandemic and can provide convincing forecasting results.

### 5.3 Ablation experiment

Ablation experiments are conducted on the model to validate further the effectiveness of the components in this work, which can evaluate the contribution of the components to the overall performance (Table 6). The attention mechanism effectively extracts the interaction information of multimodal data; however, compared with LSTM and GCN, the results are weak. The results demonstrate that both core components of the MSGCN model contribute indispensably to the stock performance. The correctness of the model decreases by % when the model is not using the LSTM module, indicating that the proposed LSTM module can effectively fit the temporal relationship between consumer sentiment and stocks. This result can help us learn the temporal features of the review text to extract consumer sentiment features on different dates. If the GCN module is not used for comment geographic information extraction, the model accuracy (expressed in terms of the rate of correct predictions) decreases by 8 % on average for the four hotels, indicating that the geographic features introduced by the GCN module primarily improve the elements missing from the time-series data. Comparing the GCN and LSTM modules, the GCN improves model performance, showing the importance of geographic features to stock prediction tasks.

**Table 6:** Ablation experiment results, these values are a percentage (For example, the higher the better).

Algorithm	TripAdvisor				Yelp			
	Crown Plaza	Marriott	Westin	Hilton	Crown Plaza	Marriott	Westin	Hilton
-Attention	55.15	56.62	55.93	56.41	54.77	55.69	54.14	55.75
-LSTM	54.32	54.96	53.87	53.20	53.12	54.96	53.46	54.82
--GCN	53.61	52.07	51.77	51.46	51.16	50.48	50.90	51.76
MSGCN	59.25	60.25	60.74	59.54	59.57	59.23	60.13	59.78

Comparing the ablation experiments on the four datasets, it is found that the two datasets exhibit a deeper dependence on the LSTM module, and the absence of the LSTM module has the most significant impact on its correctness, indicating that the Hilton dataset possesses text features with high correlation with stock movement. In contrast, the Westin dataset is more sensitive to the GCN module, implying that this dataset has more useful geographic or pictorial features. Data in Table 6 reveal the superiority of the proposed model: specifically, it outperforms traditional machine learning, and the previously processed data by extensively performing in line with the previous data with about the same mean. This indicates that the present model outperforms both data, suggesting both are useful.

**Fig. 4:**
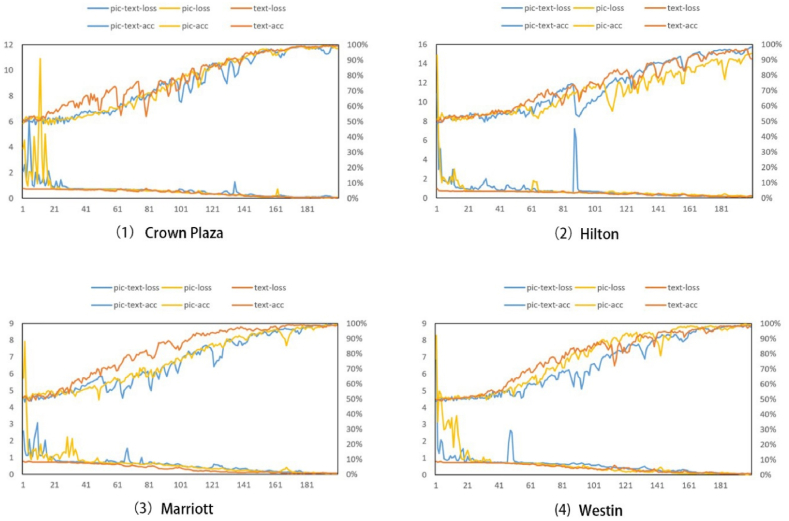
Caption: The testing process of different modalities.

Table 7 adopts data from two modalities to ensure that the model learns more multimodal features. The model testing process corresponding to the other input data is shown in Fig. 4. As can be seen from the testing data, with the increasing number of iterations, the final testing set correct rate of all models can reach more than 95 %, while the cross-entropy loss is less than 0.1, suggesting that the models can learn the features of the testing data with high accuracy for stock data prediction under 200 iterations. Regarding convergence speed, the model receiving only text input converges the fastest. Finally, the model gets multimodal information, because, at the same learning rate, the data with more features require more time cost, which is reflected in the testing process (*i.e*. slower convergence).

**Table 7:** Results for different modalities, these values are a percentage (For example, the higher the better).

Modal	TripAdvisor				Yelp			
	Crown Plaza	Marriott	Westin	Hilton	Crown Plaza	Marriott	Westin	Hilton
-text	54.68	53.56	53.02	53.82	53.29	54.10	53.44	53.45
-image	58.68	57.56	58.02	57.82	58.14	57.88	58.06	57.13
MSGCN	59.25	60.25	60.74	59.54	59.57	59.23	60.13	59.78

The test results of different models are shown in Fig. 4. The model with multimodal input shows that the highest prediction accuracy and the improvement in accuracy are noticeable. The text data are better than the input image data; furthermore, the distribution of images in the dataset is sparse and uneven because only a portion of customer reviews have images attached to it, while this portion of reviews with images follows Pareto's law that is, 20 % of the reviews contain 80 % of the images. This results in fewer vectors carrying adequate information in the image, so the model cannot efficiently glean feature information.

### 5.4 Comprehensive analysis

In our study, we employed the Multimodal Stock Graph Convolutional Network (MSGCN) due to its unique ability to handle and integrate multimodal data, specifically consumer reviews that include both text and images. The decision to use MSGCN was driven by several key strengths that make it particularly well-suited for our research objectives.

Firstly, MSGCN excels in capturing complex relationships between different types of data. Traditional models often need help with integrating heterogeneous data sources like text and images, treating them independently, which can lead to a loss of valuable contextual information. However, MSGCN embedding module effectively transforms these diverse inputs into a unified feature space, allowing for more accurate analysis. This capability is crucial for our task, where the interaction between textual sentiment and visual appeal in consumer reviews plays a significant role in influencing stock movements.

Secondly, the use of BiLSTM within the feature extraction module provides a significant advantage in modeling sequential dependencies within the text data. BiLSTM is particularly effective in understanding the context by considering both past and future word sequences, which is essential for accurately interpreting consumer sentiment. This aspect of our model is supported by the work [44], who demonstrated that BiLSTM outperforms traditional RNNs in tasks requiring deep contextual understanding.

Moreover, the GCN component offers robust capabilities in capturing relationships and dependencies among different data points. By modeling the data as a graph, where nodes represent different features extracted from the reviews, the GCN can effectively identify and leverage both local and global patterns. This is particularly advantageous in our study, as it enables the model to consider the broader context of consumer sentiment across multiple reviews, leading to more informed stock predictions. This approach aligns with the findings [45], who showed that GCNs are highly effective in tasks involving structured data relationships.

Finally, the prediction layer's linear transformation is designed to deliver high interpretability and efficiency in generating the final output. While more complex models might offer marginal gains in accuracy, they often do so at the cost of transparency and computational efficiency. Our choice of a linear layer strikes a balance, providing reliable predictions while ensuring that the model remains interpretable—a key consideration for investors and stakeholders who need to understand the rationale behind the predictions.

## 6 Discussion and conclusion

In this study, the relationship between consumer sentiment and hotel stocks is ascertained in the context of the COVID-19 pandemic: a novel hybrid model is proposed based on multimodal deep learning that incorporates GCN into multiple modal data, which can predict hotel stock prices. The hybrid model consists of three parts: pre-processing data, GCN for feature extraction, and building a predictive model. In the first stage, the multimodal data are vectorized for sentiment analysis. Second, GCN is used to analyze consumer sentiment. Finally, consumer sentiment characteristics are integrated to predict the performance of the hotel groups on the stock market before and after the pandemic. The results indicate that consumer sentiment can affect the stock price of the hotel. These findings provide theoretical and practical implications to help hotel managers prepare for future crises akin to the COVID-19 pandemic.

### 6.1 Comparative analysis

The results of our study align with previous research that has highlighted the predictive power of consumer reviews and multimodal data in financial forecasting. For example, Li et al. [46], demonstrated that textual sentiment analysis from consumer reviews could significantly influence stock market predictions, particularly in the retail sector. Similarly, Chen [47] found that incorporating visual data from product images alongside text improved the accuracy of sales forecasts, suggesting that multimodal approaches can capture a more holistic view of consumer behavior. Our model, MSGCN, builds on these foundations by integrating both text and image data through advanced techniques like BiLSTM and GCN. This approach is consistent with the findings of Hou et al. [48], who utilized a multimodal framework to predict stock prices and observed that the inclusion of image features led to better performance in volatile market conditions. Additionally, Raddant et al. [49], discussed the importance of considering both local and global dependencies in financial data, which is a principle we applied using GCN in our feature extraction module.

Furthermore, Lehrer et al. [50], emphasized the significance of contextual information in predicting market trends, particularly in sectors sensitive to consumer sentiment, such as hospitality. Our results corroborate this by showing that the combined analysis of text and image data from consumer reviews provides valuable insights into stock movements, especially during periods of economic uncertainty like the COVID-19 pandemic [51]. In summary, our findings not only validate the utility of multimodal data in financial forecasting but also contribute to the growing body of literature that underscores the need for sophisticated models that can handle complex, heterogeneous data sources. By referencing these studies, we situate our work within the broader context of financial prediction research, demonstrating the relevance and applicability of our MSGCN model to real-world market analysis.

### 6.2 Key findings

Hotel managers can utilize the insights generated by our model to understand better how consumer reviews, including sentiment and image data, influence stock performance. This information can help managers improve customer satisfaction by strategically addressing negative reviews, enhancing service offerings, or adjusting prices based on real-time feedback, leading to more robust stock performance [40]. For investors and financial analysts, the ability to incorporate non-traditional data sources like consumer reviews into stock forecasts provides a competitive advantage. Our model helps them assess the true market sentiment towards hotels beyond financial metrics, allowing for more nuanced investment strategies based on consumer behavior trends. Our findings indirectly support better decision-making in selecting hotels for consumers and travelers. As the hotel industry becomes more aware of how consumer feedback affects stock performance, they are likely to improve services, which enhances the overall consumer experience.

The success of the MSGCN model in the hospitality industry suggests that the same approach could be applied to other sectors where consumer reviews play a crucial role, such as retail, restaurants, or entertainment. This opens up new avenues for research and development in predictive modeling across different industries [52]. Our study advances machine learning by introducing a novel integration of multimodal data with spatio-temporal graph convolutional networks. This has broad implications for improving predictive models in various fields, from finance to marketing, where multimodal data is abundant but underutilized. Policymakers and regulators in the tourism and hospitality sectors could use the insights from our model to better understand the financial health of hotels, particularly in times of crisis or rapid change. This can inform policies that support the industry's resilience, such as targeted financial aid or market regulations that protect against extreme volatility.

### 6.3 Theoretical implications

This research contributes to the tourism industry in several ways:

First, the results confirm the hotel's stock market price validity based on consumer review analysis [8]. Multimodal data about text and images in online reviews provide more practical information about hotel performance. Furthermore, this study provides new insights into consumer reviews through different pre-trained models. The association between consumer sentiment and hotel stock performance has been studied and is mainly limited to exploring textual modalities. Our research compares the sentiment of the two modalities, which shows that the source of consumer sentiment is also important regarding the modality of the image. The value of the picture modality is increased, which improves hotel performance.

Second, a MSGCN that extracts the consumer's features is developed. Advanced pre-training models are used to vectorize the elements for text and pictures. An MSGCN is then utilized to construct hotel location information, which improves the accuracy of hotel stock price predictions. This study provides management guidelines for finance managers, which can be extended to many fields.

Thirdly, time series are used to compare the performance of consumer sentiment on hotel stocks before and after the outbreak of the COVID-19 pandemic. This study involves a systematic investigation of the changes in consumer sentiment in hotels before and after the pandemic, which examines the impact of consumer sentiment on stock returns. Thus, this research provides tourism firms with information about how best to manage their stock performance during the pandemic. It also strengthens the cooperation between the stakeholders involved in the hotel management process, thereby improving financial performance.

Additionally, the findings provide hotel managers with valuable ideas to help them cope with adversity. The current situation demonstrates an urgent need to move from crisis management to improving adaptive learning from disruption. This paper [52] strengthened stakeholder collaboration in the hotel management process.

In summary, this study contributes to the literature by demonstrating the effectiveness of multimodal deep learning and GCN in sentiment analysis. These techniques allow for a more comprehensive and accurate assessment of consumer sentiment, which is crucial for understanding its impact on financial markets. This research provides a foundation for future studies to explore even more advanced AI techniques in sentiment analysis, potentially leading to new methodologies and frameworks. By integrating spatio-temporal features into sentiment analysis, this study addresses a critical gap in the literature. This approach provides a more holistic view of how consumer sentiment varies across locations and times, and how these variations influence market dynamics. This contributes to a deeper understanding of the complex interplay between consumer behavior, geographic factors, and financial outcomes, paving the way for more nuanced research in tourism and monetary economics.

### 6.4 Practical implications

The explosive growth in the volume of consumer review data has led more hotel managers to pay attention to consumer sentiment. However, hotel stakeholders are only concerned with the modality of the text. An appropriate method is used to analyze consumer reviews. Our research provides valuable sentiment analysis methods. This provides some insight for hotels or financial institutions on leveraging consumer reviews. Stakeholders should allocate resources to analyze consumer reviews regularly. Our research helps hotel managers better understand consumer sentiment in the stock market, and these findings provide some management recommendations.

First, keeping up to date with the latest artificial intelligence technologies: our proposed MSGCN is influential in optimal feature extraction of hotel location information. Coupled with the time-series information of consumer reviews, the accuracy of our proposed model is significantly improved compared to machine learning and deep learning. Therefore, hotel stakeholders can monitor their stock performance using this framework to understand consumer sentiment media. Additionally, using customer information, stakeholders can leverage information that supports other financial services, such as hotel equity analysis.

Second, the response strategies of hotel managers against the pandemic should involve comparing hotel stock data before and after the pandemic. The apparent development of the pandemic has a direct impact on consumer sentiment. The performance of hotels during the pandemic is significantly weaker than before it. Hotel managers can strategically respond to the COVID-19 pandemic. Hoteliers must face the challenges of the current crisis with limited time and information, while preparing for an upturn in an atmosphere of uncertainty.

The study highlights the importance of ethical considerations in applying AI and sentiment analysis. Policymakers can use these findings to refine AI regulations, ensuring that they promote transparency, fairness, and accountability in AI-driven sentiment analysis. This could involve setting standards for data usage, algorithmic transparency, and consumer protection, thereby fostering trust in AI applications [53]. The insights from this study could inform economic policies aimed at supporting the tourism industry, particularly in the aftermath of crises like the COVID-19 pandemic. Policymakers could leverage sentiment analysis to gauge the public's perception of tourism-related policies and their effectiveness. This, in turn, can guide policy adjustments to better align with consumer expectations and enhance the tourism sector's resilience.

The study underscores the power of consumer voices in shaping market outcomes. The sentiment expressed in online reviews can significantly influence stock prices, affecting the broader economy. This highlights the growing importance of consumer feedback in the digital age. As consumers become more aware of their reviews' impact, they may be more motivated to provide honest and constructive feedback, ultimately leading to better products and services. The study's emphasis on ethical AI aligns with societal concerns about privacy, fairness, and transparency in AI applications [54]. Society can benefit from a more responsible use of AI, where consumers are protected from potential biases and data misuse. This reinforces the need for continuous dialogue between AI developers, regulators, and the public to ensure that AI technologies serve the common good.

### 6.5 Limitations and future research

Although this study provides specific insights, it has limitations: first, using previous research, TripAdvisor and Yelp are two data sources chosen since they are consumer review sites. However, future research should include more travel sites to understand this phenomenon better, such as Ctrip, Qunar, Booking, and others.

Secondly, this research adopts an MSGCN for modeling, which can effectively extract features but does not utilize the latest deep learning algorithms. New frameworks, such as contrastive learning and prompt learning, will be considered in future work.

Third, only two modalities are selected to represent consumer sentiment. Multivariate techniques are used to determine their correlations in the future more thoroughly. Empirically examining the impact of these variables on hotel stocks will be helpful.

The main limitation of this study is that it is only a partial, unpredictable view of the pandemic. The corresponding multimodal time-series dataset is constructed from two data sources. Then, BERT and ResNet extract text and image features; the stock prediction model based on LSTM and GCN is constructed for multimodal data input. Finally, comparing baseline models, ablation experiments, and multi-modal comparisons can verify this experimental model's progressive nature and effectiveness. Survey results yield important insights to guide hotel managers in responding to the current pandemic and preparing for the near future.

Moreover, conduct longitudinal studies that track consumer sentiment and hotel stock performance over an extended period [55]. Future studies could examine how external factors such as government policies, environmental changes, or macroeconomic conditions interact with consumer sentiment to influence hotel stock performance. This would provide a more holistic understanding of the factors that drive stock performance beyond consumer sentiment alone. For example, how do travel restrictions, environmental regulations, or economic stimulus package changes impact consumer sentiment and, subsequently, hotel stocks?

The COVID-19 crisis allows hotel managers to investigate consumer sentiment despite the uncertainty created by the problem and its impact on the long-term perspective of consumer sentiment on potential risks to hotel companies, which adjust business models promptly [56]. The findings design a better understanding of consumer purchase intentions, which helps improve service quality and strategic positioning. Therefore, shaping the hotel industry's response to the COVID-19 crisis effectively targets potential shocks and catastrophic events affecting the future of the hotel industry.

## 7 Conclusions

The corresponding multimodal time-series dataset is established from two data sources: BERT and ResNet are then utilized to extract the text features and image features, and the stock-price prediction model based on LSTM and GCN is constructed for multimodal data input. Finally, comparing baseline models, ablation experiments, and multi-modal comparisons can verify the progressive nature and effectiveness of the proposed model. Survey results provide important insights to guide hotel managers in responding to the current pandemic crisis and preparing for the near future. The COVID-19 crisis offers an opportunity for hotel managers to investigate consumer sentiment despite the uncertainty created by the problem and its impact on the long-term perspective of consumer sentiment on potential risks to hotel companies, which adjust business models promptly. The findings design a better understanding of consumer purchase intentions, which helps improve service quality and strategic positioning. Therefore, shaping the hotel industry's response to the COVID-19 crisis effectively targets potential shocks and catastrophic events affecting the future of the hotel industry.

## CRediT authorship contribution statement

**Yang Liu:** Writing – original draft, Software, Methodology, Data curation. **Lili Ma:** Supervision, Investigation.

## Data availability

Data will be made available on request.

## Funding

This work was supported by the 10.13039/501100001809National Natural Science Foundation of China (No. 72204190), the Research Foundation of 10.13039/501100002338Ministry of Education of China (No. 22YJZH114), the Capital Circulation Industry Research Base of 10.13039/501100005706Beijing Technology and Business University (SDLT202204), Scientific research project of the National Language Commission of China (No. YB145-74), Thanks for the support by iSchools Research Grants and the Fundamental Research Funds for the Central Universities.

## Declaration of competing interest

The authors declare the following financial interests/personal relationships which may be considered as potential competing interests:Yang Liu reports financial support was provided by Wuhan University. Reports a relationship with that includes:. Has patent pending to. If there are other authors, they declare that they have no known competing financial interests or personal relationships that could have appeared to influence the work reported in this paper.
